# Category verbal fluency performance may be impaired in amnestic mild cognitive impairment

**DOI:** 10.1590/s1980-57642008dn10200008

**Published:** 2007

**Authors:** Márcio Luiz Figueredo Balthazar, Fernando Cendes, Benito Pereira Damasceno

**Affiliations:** 1 Postgraduate Neurologist. Department of Neurology, Medical School, State University of Campinas - SP, Brazil.; 2 Associate Professor. Department of Neurology, Medical School, State University of Campinas - SP, Brazil.; 3 Professor, Department of Neurology, Medical School, State University of Campinas - SP, Brazil.

**Keywords:** verbal fluency, mild cognitive impairment, Alzheimer disease, neuropsychological tests

## Abstract

To study category verbal fluency (VF) for animals in patients with amnestic mild cognitive impairment (aMCI), mild Alzheimer disease (AD) and normal controls.

**Method:**

Fifteen mild AD, 15 aMCI, and 15 normal control subjects were included. Diagnosis of AD was based on DSM-IV and NINCDS-ADRDA criteria, while aMCI was based on the criteria of the International Working Group on Mild Cognitive Impairment, using CDR 0.5 for aMCI and CDR 1 for mild AD. All subjects underwent testing of category VF for animals, lexical semantic function (Boston Naming-BNT, CAMCOG Similarities item), WAIS-R forward and backward digit span, Rey Auditory Verbal Learning (RAVLT), Mini-Mental Status Examination (MMSE), and other task relevant functions such as visual perception, attention, and mood state (with Cornell Scale for Depression in Dementia). Data analysis used ANOVA and a post-hoc Tukey test for intergroup comparisons, and Pearson’s coefficient for correlations of memory and FV tests with other task relevant functions (statistical significance level was p<0.05).

**Results:**

aMCI patients had lower performance than controls on category VF for animals and on the backward digit span subtest of WAIS-R but higher scores compared with mild AD patients. Mild AD patients scored significantly worse than aMCI and controls across all tests.

**Conclusion:**

aMCI patients may have poor performance in some non-memory tests, specifically category VF for animals in our study, where this could be attributable to the influence of working memory.

## Abstract (pt)

Estudar a fluência verbal (FV) para a categoria animais no comprometimento cognitivo leve amnéstico (aCCL), doença de Alzheimer (DA) leve e controles normais.

**Método:**

Incluímos 15 pacientes com DA leve, 15 com aCCL e 15 controles normais, usando os critérios DSM-IV, NINCDS-ADRDA e CDR 1 para DA, e os do International Working Group on Mild Cognitive Impairment, e CDR 0,5 para aCCL. Todos os sujeitos passaram por avaliação da FV para a categoria animais, função léxico-semântica (Teste de nomeação de Boston - TNB, item de Similaridades do CAMCOG), extensão de dígitos direto e indireto do WAIS-R, aprendizado auditivo-verbal de Rey (TAAVR), Mini-Exame do Estado Mental (MEEM), e de outras funções (contraprovas) capazes de influenciar nestes testes, como percepção visual, atenção e estado de humor (este com a Escala Cornell para Depressão em Demência). A análise dos dados usou o teste de análise de variância (ANOVA) seguido do teste de Tukey post hoc para comparações entre os grupos e o coeficiente de Pearson para correlação entre testes e contraprovas (nível de significância p<0,05).

**Resultados:**

Os pacientes com aCCL tiveram performance inferior à dos controles nos testes de FV para animais e na extensão de dígitos indireta do WAIS-R. Pacientes com DA leve tiveram performance inferior à de sujeitos com aCCL e controles em todos os testes.

**Conclusão:**

Pacientes com aCCL tiveram desempenho rebaixado em testes de fluência verbal para animais, o que pode ter sido influenciado pela memória operacional.

Mild cognitive impairment (MCI) is a clinical entity in patients with objective cognitive problems (most often episodic memory) but without impairment in daily life activities,^1^ having a greater likelihood of transforming into dementia, most often Alzheimer disease (AD), than in the normal population.^2^ MCI can be classified according to the clinical presentation of symptoms into amnestic MCI (aMCI), multiple domain or single non-memory domain MCI.^1,2^ Thus, by definition, aMCI presents with exclusive memory deficit, sparing other cognitive domains such as language, visuospatial perception or executive functions. Nonetheless, aMCI individuals may present some non-memory-related poor performance in specific neuropsychological tests, following a pattern similar to AD,^3^ and continue to be classified as amnestic rather than multiple domains MCI. This classification is based on the clinical judgment that poor performance in one test is not enough to consider an entire cognitive domain as impaired.

Verbal fluency (VF) for animal’s names is a simple and widely used task that can reveal impairment in early phases of AD,^4^ where a recent study points to impairment even in aMCI.^3^ Category VF involves several cognitive aspects, such as semantic knowledge, executive function and working memory. Henry et al. suggested that verbal fluency is “an excellent way of evaluating how subjects organize their thinking and ability to “organize output in terms of clusters of meaningfully related words”.^5^

Our aim was to compare verbal fluency (category: animals) in healthy controls and patients diagnosed as aMCI and mild AD, hypothesizing that these two groups of patients have similar performance, because impairment of this function is common even in early stages of AD.

## Methods

We studied 45 subjects, comprising 15 with aMCI and 15 with mild AD attended at the Unit for Neuropsychology and Neurolinguistics (UNICAMP Clinic Hospital), along with 15 controls. Routine laboratory examinations for dementia assessment (including B12 and folate dosage, sorology for syphilis, thyroid hormones) and brain computed tomography were carried out in all patients. The local ethics committee approved this research.

MCI in our clinic is a clinical diagnosis carried out by trained neurologists using a standardized mental status battery and was based on the following criteria of the International Working Group on Mild Cognitive Impairment:^1^

(i) the person is neither normal nor demented;
(ii) there is evidence of cognitive deterioration shown by either objectively measured decline over time and/or subjective report of decline by self and/or informant in conjunction with objective cognitive deficits; and
(iii) activities of daily living are preserved and complex instrumental functions are either intact or minimally impaired.

We included only patients older than 50 years who had a CDR (Clinical Dementia Rating)^6^ of 0.5. This classification was performed by using a semi-structured interview.

For probable AD diagnosis, we used the criteria of the National Institute of Neurological and Communicative Disorders and Stroke (NINCDS) and Alzheimer’s Disease and Related Disorders Association (ADRDA)^7^, including only patients classified as CDR 1. Exclusion criteria were history of other neurological or psychiatric diseases, head injury with loss of consciousness, use of sedative drugs within 24 hours of the neuropsychological assessment, drug or alcohol addiction and prior exposure to neurotoxic substances. The control group consisted of subjects with CDR 0 and no previous history of neurological or psychiatric disease, or memory complaints.

Neuropsychological evaluation comprised the following tests:

1. Verbal fluency (VF) for animals’ category (the score was the total number of different animal names given the by patient in one minute).
2. Mini Mental Status Examination (MMSE),^8^ Brazilian version.
3. Episodic memory was evaluated using the Rey auditory verbal learning test (RAVLT).^9^
4. Boston Naming Test (BNT- translated and culturally adapted version for Brazilian population by Dr. Cândida Camargo – Psychiatry Institute, Medicine School, University of São Paulo).^10^ The BNT score was the sum of spontaneous correct responses plus correct responses following a semantic cue.
5. CAMCOG’s subscale of similarities between pairs of nouns.^11^ The patients were asked “ In what way are they alike?” for the pairs apple/banana, chair/table, shirt/dress and animal/vegetable. The score was calculated as the number of correct responses (zero to two for each pair; maximum score 8).
6. Visual perception subtests of Luria’s Neuropsychological Investigation^12^ (LNI; maximum score 20).
7. Attention: The forward and backward digit span subtest of WAIS-R.^13^
8. Cornell Scale for Depression in Dementia^14^ (CSDD).

Data analysis by means of Systat software used ANOVA and a post-hoc Tukey tests for intergroup comparisons of demographic and cognitive scores, as well as Pearson coefficient for correlation between tests. Statistical significance considered was p<0.05.

## Results

The results of demographic data are shown in Table 1 and neuropsychological evaluation in Table 2. aMCI subjects were similar to controls in age (p=0.576) and education (p=0.483). aMCI subjects performed similar to controls in CAMCOG’s item of similarities (p=0.789) and Boston Naming Test (p=0.582) but performed worse than controls in verbal fluency (p<0.001), MMSE (p=0.034), backward digit span (p<0.05), delayed recall (p<0.001) of RAVLT, CAMCOG’s item of similarities (p=0.789) and Boston Naming Test (p=0.582).

**Table 1:** Demographics results of amnestic mild cognitive Impairment (AMCI), Alzheimer disease (AD), and normal control subjects.

	AD	MCI	Controls	
	(n=15) Mean±SD	(n=15) Mean±SD	(n=15) Mean±SD	p value for intergroup effect
Age	75.66±7.65	66.26±10.27	69.40±7.28	AD x MCI: p=0.012 AD x Controls: p=0.121 MCI x Controls: p=0.576
Education (years)	4.86±4.76	5.93±4.18	6.73±3.59	p=0.483

**Table 2:** Neuropsychological results of amnestic mild cognitive Impairment (AMCI), Alzheimer disease (AD), and normal control subjects.

	AD	MCI	Controls	
	(n=15) Mean±SD	(n=15) Mean±SD	(n=15) Mean±SD	P value intergroups
MMSE	22.53±3.06	26.86±2.50	29.06±0.70	AD x MCI: p<0.001 AD x Controls: p<0.001 MCI x Controls: p=0.034
VF	10.20±3.44	13.86±3.85	19.46±3.31	AD x MCI: p=0.019 AD x Controls: p<0.001 MCI x Controls: p<0.001
BNT	38.73±8.64	51.06±7.78	53.66±4.11	AD x MCI: p=0.001 AD x Controls: p<0.001 MCI x Controls: p=0.582
A7- RAVLT	1.00±1.25	4.26±2.54	9.40±3.20	AD x MCI: p=0.002 AD x Controls: p<0.001 MCI x Controls: p<0.001
Similarities	4.86±1.80	7.00±1.19	7.33±1.04	AD x MCI: p<0.001 AD x Controls: p<0.001 MCI x Controls: p=0.789
fDS	4.73±1.03	4.60±0.82	4.93±0.79	p=0.583
bDS	3.13±0.51	3.13±0.99	3.93±1.09	AD x MCI: p=1.000 AD x Controls: p<0.05 MCI x Controls: p<0.05
Visuo-spatial LNI	17.33±1.39	18.80±1.01	18.66±1.11	AD x MCI: p=0.004 AD x Controls: p=0.01 MCI x Controls: p=0.949

MMSE, mini-mental status examination; fDS, forward digit span; bDS, backward digit span; VF, verbal fluency; BNT, Boston naming test; A7- RAVLT, delayed recall of Rey auditory verbal learning test.

AD patients were older than aMCI (p=0.012) but not control subjects (p=0.121). The educational level of the AD group was lower than that of controls (though not statistically significant). These patients scored lower than controls and aMCI subjects on all tests, except the forward digit span. The cognitive performance of mild AD was worse than aMCI, which in turn was poorer than controls.

The analysis of relationships between tests in the groups showed statistically significant correlations only between VF and RAVLT delayed recall in the AD group (r=0.545; p<0.05) and between VF and BNT in the aMCI group (r= 0.540; p<0.05). In AD group, FV tended to correlate to BNT, but not reaching statistical significance (p=0.066). Scores on the Cornell Scale for Depression did not correlate to any of the cognitive tests: F (2,42)=0.929; p=0.403.

## Discussion

Our findings showed that aMCI patients performed worse than controls but better than mild AD on the category VF task. This task involves not only speed and ease of word production, but also lexical-semantic field selection, executive function and working memory, in keeping track of what words have already been said. Some authors have found poor performance on category VF in MCI patients, and have interpreted this finding as a degradation of semantic networks.^15-17^ We suggest that working memory and attention, rather than semantic or executive function deficits, may have influenced VF in our patients, since aMCI subjects had significantly lower scores on backward digit span test yet normal performance in semantic and executive tasks (neither anamnesis nor objective cognitive tests used in our diagnostic process showed executive dysfunction in any patients classified as aMCI). In fact, Perry et al.^18^ have shown that deficits in attention are more prevalent than deficits in semantic memory in early AD. Similarly, our results on lexical semantic tests such as BNT and CAMCOG’s similarities, showed no difference between aMCI and controls. Thus, our findings suggest that semantic knowledge is not impaired and cannot explain the poor performance of this group of patients in category VF.

AD patients’ low VF was correlated to their impaired RAVLT delayed recall. A plausible explanation for this finding could be that our VF task partly depends on active retrieval (lexical-semantic selection) of animals’ names from long-term declarative memory, also the case in the RAVLT delayed recall task. On the other hand, it is difficult to explain why VF was correlated to BNT in the aMCI group, since this group performed as well on the BNT as did controls. Nevertheless, the fact that FV was correlated to BNT in the aMCI group and also tended to correlate in the AD group, suggests that both groups may have impairment of some linguistic competence involved in lexical-semantic selection, although this was not specifically tested in our study.

We have found that aMCI patients may have poor performance in some non-memory tests, specifically category VF for animals, and that this could be attributable to the influence of working memory. However, further studies using more comprehensive testing of VF, including a phonemic task, as well as more specific tests for executive function and lexical-semantic selection in a larger sample are needed for more robust conclusions to be drawn.

## Figures and Tables

**Figure 1:**
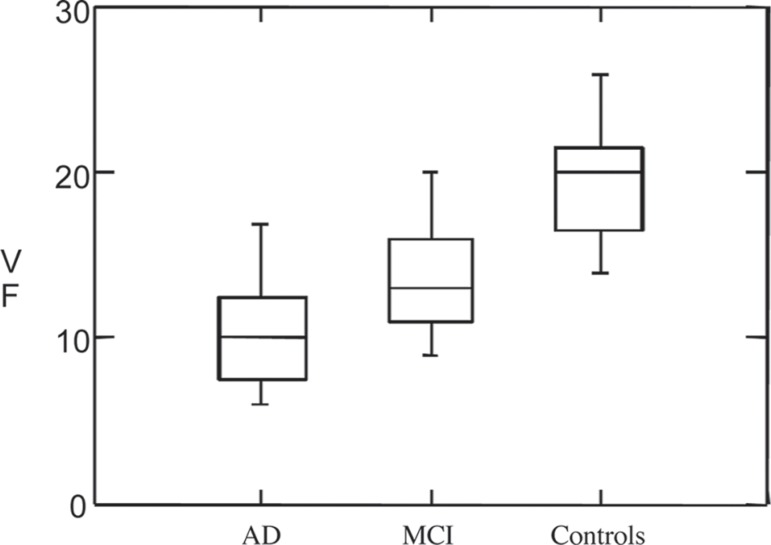
Distribution of verbal fluency scores of AD, aMCI and control subjects.
